# Effects of postural intervention using a *risshin* chair on physical posture, class time perception, and subjective well‐being in junior high school students

**DOI:** 10.1111/bjep.70087

**Published:** 2026-04-20

**Authors:** Yusuke Murakami, Takashi Akiyama, Genji Sugamura

**Affiliations:** ^1^ Department of Psychology, Faculty of Letters Kansai University Suita‐shi Japan; ^2^ Psychology Major, Faculty of Sociology Kansai University Suita‐shi Japan

**Keywords:** embodied cognition, junior high school, learning environment, posture education, posture regulation, subjective well‐being

## Abstract

**Background:**

Research based on embodied cognition has demonstrated that physical posture, particularly an upright seated posture, can influence emotions and cognition. However, although students spend most of the day sitting in classrooms, intervention research on upright seated postures suitable for learning environments is limited.

**Aims:**

We aimed to examine the effects of postural intervention via a ‘Zazen‐like upright‐support seating furniture’ (a *risshin* chair) on physical posture, class time perception, positive affect, and daily sense of fulfilment in school in a junior high school setting.

**Sample:**

The participants were 48 second‐year junior high school students (25 girls and 23 boys; *M*_age_ = 13.39 years, *SD* = .49) from two classes of a single grade at a public school in Japan.

**Methods:**

Using a within‐participants design, students alternated between sitting in regular and *risshin* chairs in an A‐B‐A‐B‐A order for approximately five weeks. At the end of each week, they completed a self‐report questionnaire during the final homeroom session on every Friday.

**Results:**

Bayesian linear mixed‐effects modelling revealed significant effects of the *risshin* chair on posture‐related outcomes, such as autonomous posture improvement, straight back, and tension release. Bayesian mediation analysis with linear mixed‐effects modelling further showed that the *risshin* chair reduced time‐related distracted thoughts, increased pleasantness, and enhanced daily school fulfilment, with these effects mediated via improved straight‐back posture.

**Conclusions:**

Although short‐term, the *risshin* chair improved specific aspects of students' physical posture, class time perception, and subjective well‐being during school compared with regular chairs.

## INTRODUCTION

Over the past few decades, cognitive science and psychology have increasingly emphasized the roles of the body, artefacts and social contexts in cognition. This research trend is often discussed within the framework of ‘4E cognition’ (embodied, embedded, enacted and extended; Newen et al., 2018), which challenges the traditional view of cognition as internal information processing. Instead, psychological processes emerge from interactions between bodily actions and the environment. In educational psychology, the 4E approach has demonstrated that embodied interactions, such as gestures and tangible actions, function as external cognitive tools that stabilize attention and scaffold reasoning (Pouw et al., 2014; Wang et al., 2025). Furthermore, recent studies have revealed that embodied experiences facilitate conceptual understanding through socially shared engagement (Ferreira & Ineson, 2026).

From the perspective of 4E cognition, a learner's seated posture can be viewed as an embodied dimension of self‐regulation rooted in physiological arousal, affect and attention. In other words, sustained bodily states such as posture function as a somatic foundation for maintaining and enhancing psychological engagement. Research has increasingly demonstrated that postural and psychological states are dynamically interrelated. For instance, slumped postures may exacerbate negative affect (Dehcheshmeh et al., 2024). Recent experimental evidence indicates that posture serves as a somatic affordance for emotion regulation. In this context, an affordance refers to the fit between an individual's goals (e.g., emotion regulation) and the available means within the current situation (e.g., bodily posture) to achieve those goals (Veenstra et al., 2017). Specifically, a stooped posture impedes recovery from negative moods and increases negative thoughts (Veenstra et al., 2017), which may consequently undermine sustained engagement in learning. Therefore, classroom chairs, which directly influence learners' seated postures, can be conceptualized as somatic scaffolds or extended cognitive supports. Recent studies have confirmed that specific seat configurations significantly affect performance in concentrated learning tasks (Chen & Tsai, 2024). These findings indicate the need for further experimental research to elucidate the causal impact of postural interventions on students' academic engagement and psychological well‐being.

In Japan, such an emphasis on posture is deeply rooted in the culture. Teachers have often intuitively emphasized ‘straightening the back’ to enhance students' focus and emotion regulation. Historically, stable and upright postures have been prioritized in mental discipline practices, such as Zen meditation and martial arts (Yuasa, 1993). Particularly within Buddhist traditions, early canonical texts describing mindfulness of breathing identify maintaining an erect body (‘*ujuṃ kāyaṃ paṇidhāya*') as a fundamental preparatory condition for meditation practice (Anālayo, 2019). Similarly, contemplative procedures in Tibetan Buddhism such as loving‐kindness meditation explicitly instruct practitioners to adopt an upright seated posture (Nash et al., 2013). These traditions collectively indicate that the upright posture functions as a foundational bodily basis for contemplative practice across diverse Buddhist traditions. In Japan, this concern remains relevant even today. For instance, the Japanese Ministry of Education, Culture, Sports, Science and Technology (MEXT, 2022) has highlighted the need for environmental adjustments to prevent postural deterioration during Information and Communication Technology (ICT)‐based learning. These cultural and administrative insights align with empirical findings that a temporary upright posture aids cognitive and emotional functioning (e.g., Awad et al., 2021; Inagaki et al., 2018) and correlates with academic outcomes such as exam scores (Weisfeld & Beresford, 1982). However, educational psychology has yet to fully elucidate the empirical pathways or cumulative impact of supporting student postures over extended periods in actual school settings. To address these gaps, we examined how modifying the physical environment using the *risshin* chair facilitates embodied engagement and daily school fulfilment. We conceptualized postural support as a somatic scaffold for embodied self‐regulation using continuous intervention in junior high school students. This approach clarifies how environmental design can anchor attention and enhances subjective well‐being, thereby contributing to a broader framework of student adaptation.

### Impact of posture on cognition and emotion

There is a lack of systematic reviews on how upright posture influences learners' psychological outcomes. Grounded in theories of embodied cognition (Winkielman et al., 2015) and embodied psychology (Haruki & Yamaguchi, 2016), this study posits that bodily sensations and sustained orientations, such as posture, shape mental information processing. From the 4E perspective, an erect or upright posture can enhance a vivid sense of awareness (Haruki & Suzuki, 1994), facilitate a more positive interpretation of ambiguous stimuli (Miragall et al., 2020) and increase the perceived ease of cognitive tasks (Peper et al., 2018). Moreover, maintaining an upright posture has been shown to improve the processing speed in attention tasks (Awad et al., 2021) and intelligence tests (Inagaki et al., 2018).

A classical account of psychological mechanism explaining how posture influences cognition and emotion is provided by self‐perception theory. This theory suggests that individuals understand their own internal attitudes and emotions by observing their own overt behaviour and the situational context in which it occurs, much like an external observer (Bem, 1972). Empirical evidence supports this, demonstrating that adopting an upright seated posture under stress can maintain higher self‐esteem and improve mood compared to adopting a slumped posture (Nair et al., 2015).

Biomechanically, posture is defined as the ‘position and relative arrangement of the body parts’ (Canales et al., 2010, p. 376). Suboptimal posture stems from a defective relationship among body segments, which increases tension across the supporting musculoskeletal structures (Canales et al., 2010). To prevent instability, misalignment forces the body to engage in compensatory efforts across different segments to sustain physical equilibrium (Guimond & Massrieh, 2012). In adolescents, improper posture, represented by a slumped posture, is significantly associated with lower self‐efficacy and poorer back muscle endurance (Smith et al., 2010). Consequently, providing an environmental affordance that scaffolds optimal posture may reduce this hidden compensatory workload, thereby freeing up the attentional resources necessary for deep engagement and self‐regulation in classrooms.

Such upright trunk posture is generally associated with positive effects on mental state. However, empirical studies examining the effects of such posture in actual school settings remain scarce. Furthermore, such postures are temporarily manipulated under experimental conditions, making it unrealistic to expect children and adolescents to maintain them for extended periods in classrooms. A thoracic upright posture involving slight shoulder blade retraction and lumbar extension, which activates more spinal erecting muscles compared with a pelvic upright posture (O'Sullivan et al., 2006), may inadvertently increase muscle tension and both physical and mental stress.

### Intervention studies using posture‐supporting seating

Various approaches exist to improving learners' posture (Mori, 1983; Otsui et al., 2006). A beneficial approach includes introducing seating aids to assist with prolonged sitting. Among these aids, we focused on the ‘*risshin* (立身) chair’, a device supported by empirical research integrating postural studies and embodied psychology and traditional Zen meditation (Haruki, 2006; Sugamura, 2016). This chair promotes an upright ‘*risshin* posture’ (Murakami et al., 2022), which keeps the pelvis straight without excessive forward or backward tilt, maintains moderate spinal elongation, releases shoulder tension and positions the skull naturally on the spinal axis. Spinal stress increases as the head tilts forward, while good posture with ears aligned to the shoulders and shoulder blades retracted, minimizes this stress and is most efficient for the spine (Hansraj, 2014). The *risshin* posture is ideal for learning as it helps reduce such loads. In the 4E framework, this chair acts as an extended regulatory resource that stabilizes a learner's physiological foundation.

Empirical evidence links somatic stabilization to cognitive‐affective outcomes. Near‐infrared spectroscopy has shown that the *risshin* posture increases activation in the dorsolateral prefrontal cortex during cognitive tasks compared to slumped sitting (Sugamura et al., 2007). Elementary school students have reported feeling more relaxed yet energetic in *risshin* chairs (Sugamura et al., 2016). Continuous use, combined with morning meditation, can enhance posture awareness, reduce irritability and promote calmness, with teachers noting higher student motivation and concentration (Haruki, 2006, 2011; Sugamura, 2016). In university students, *risshin* chairs foster higher emotional engagement and relaxation while reducing bodily discomfort (Murakami & Sugamura, 2017). Although a follow‐up study (Murakami & Sugamura, 2019) found no significant differences in emotional engagement, students reported lower engagement when experiencing physical discomfort and fatigue in class. Recent studies have explored detachable cushion‐type *risshin* seats. Laboratory results using a force plate indicated that these seats suppress anterior–posterior and fast‐posture oscillations (Sugamura et al., 2023). Moreover, a one‐week intervention with high school students demonstrated improved posture during classes, fast class time perception, enhanced mental health and a sense of meaningfulness in school life (Murakami et al., 2022). The free‐response data highlighted perceived improvements in posture, relief from fatigue and physical discomfort, and heightened concentration during the classes. Taken together, these findings suggest that physical alignment facilitated by the *risshin* chair functions as a somatic scaffold that optimizes cognitive resources and fosters higher‐order mental engagement.

### Issues in prior research

Intervention studies using *risshin* chairs have encountered several challenges. First, Murakami et al. (2022) restricted the study to only second‐year high school students in advanced classes. Hence, there is a need for intervention studies in public middle schools that comprise learners with diverse academic motivations and abilities.

Second, Murakami et al. (2022) employed an A‐B‐A design that involved a single period of intervention with *risshin* chairs. This design may have overly piqued students' interest owing to the use of an experimental apparatus different from regular chairs, potentially positively biasing their overall responses. We adopted an A‐B‐A‐B‐A design to verify the reproducibility of the effects of *risshin* chairs intervention.

Furthermore, we expanded the measurement items to better capture embodied and active learning processes. Beyond simply maintaining a ‘good posture’, we added posture‐related items such as ‘straight back’ and ‘tension release’ to assess whether the chair truly functioned as a somatic scaffold. In addition, we included a question regarding whether the students sat deeply in the chair and whether they attempted to improve their posture autonomously through the use of support. For concentration, we moved beyond simple time perception (i.e., whether class time felt faster) to include ‘time‐related distraction’ (Rutrecht et al., 2021), reflecting the extent to which learners remain situated in their tasks. As a state of flow, characterized by altered time perception, is positively associated with academic performance (Jinmin & Qi, 2023; Leigh et al., 2021), these measures provide deeper insights into how postural interventions anchor attention in educational settings. These new items allow for a more comprehensive understanding of how physical affordances support the embodied foundations of student engagement.

### Mechanisms linking posture to class time perception

Subjective time perception—the ability to estimate and experience time intervals (Vignaud et al., 2025)—is not a mere reflection of objective duration but varies with a learner's psychophysiological state. In classroom settings, when class periods are fixed, students may perceive time as passing quickly during high engagement or dragging slowly during boredom or disengagement (Murakami et al., 2022). Temporal distortion is governed by attention allocation and physiological arousal. According to the pacemaker–accumulator mechanism and attentional gate theory, an accelerated internal clock (due to high arousal) or increased attention towards temporal monitoring (due to task disengagement) leads to an overestimation of duration (van der Mijn & van Rijn, 2021; Zakay & Block, 1997). Crucially, bodily experiences such as stress, discomfort, or pain can capture attentional resources and increase sympathetic nervous system arousal, thereby dilating subjective time (Rey et al., 2017; Vignaud et al., 2025). Neuroscientific evidence suggests the anterior insular cortex plays a key role in this process by integrating interoceptive body information with the subjective experience of time (Craig, 2009).

Conventional school chairs often cause physical discomfort, which may interfere with concentration (Murakami et al., 2022) and influence time perception both physically and cognitively. Unpleasant bodily sensations can induce negative arousal and accelerate internal pacemakers. Furthermore, voluntary postural maintenance requires continuous motor control and cognitive effort, which imposes a ‘hidden’ workload. Although a self‐sustaining upright posture can increase discomfort and cognitive load, appropriate postural support has been shown to reduce this burden and enhance EEG activity associated with sustained attention (Jung & Kang, 2024). Within the attentional gate framework, such somatic demands may widen the gate for temporal pulses, causing time to feel slower.

By providing a somatic scaffold, the *risshin* chair is expected to alleviate this compensatory workload. This passive support stabilizes bodily states and liberates attentional resources from postural regulation and time monitoring, redirecting them towards learning activities. Consequently, the *risshin* chair may facilitate a state of flow in which temporal awareness diminishes, and class time is perceived as passing more quickly. Therefore, subjective time perception may serve as an indirect indicator of attentional engagement, reflecting the extent to which students remain immersed in classroom activities.

### Mechanisms linking posture to subjective well‐being

This study conceptualized subjective well‐being as a multi‐dimensional composite. Subjective well‐being is generally defined as an individual's affective and cognitive/evaluative judgements about their life (Diener, 2012). Recent comprehensive efforts to organize the psychological aspects of well‐being have specified that emotional well‐being encompasses both experiential and reflective features (Park et al., 2023). Specifically, it includes the affective quality of moment‐to‐moment and everyday experiences (hedonic/affective) as well as the sense of meaning, life satisfaction and ability to pursue goals (eudaimonic/cognitive). Based on this framework, we assessed student well‐being using two distinct dimensions tailored to the school context. Positive affect (pleasantness and relaxation) was used to capture the experiential quality of emotion during school hours. Second, daily school fulfilment was utilized to represent the reflective appraisal of meaning within the daily educational context, rather than broader, abstract philosophical reflections.

According to the theories of embodied cognition and bodily feedback accounts, bodily states constitute integral components of emotional representations. Emotional experiences are encoded through interactions among modality‐specific sensory, motor and affective systems and are later reactivated during emotional processing (Niedenthal, 2007). Consequently, adopting bodily states associated with particular emotions, such as an upright posture, may partially re‐enact these representations and elicit corresponding affective experiences. Studies show that adopting an upright posture increases positive affect compared to a slumped posture (Awad et al., 2021) and enhances states such as vitality and pleasure relative to a neutral posture (Inagaki et al., 2018). The emotional effects of posture have also been interpreted in terms of interoceptive processes, such as access to internal bodily signals. In particular, an upright posture has been linked to enhanced access to interoceptive cues associated with calmness or vitality, which may contribute to more positive affective states (Lin et al., 2026). Postural support provided by *risshin* chairs has been reported to induce a ‘relaxed yet energetic’ state in the short term (Sugamura et al., 2016) and to enhance daily positive affect while reducing physical discomfort over the medium term (Murakami et al., 2022). Thus, the optimized maintenance of an upright posture may partially re‐enact bodily components of positive emotional states, and the repeated experience of such positively valenced bodily states supported by postural stability may exert a sustained positive influence on the affective components of well‐being.

Furthermore, the embodied regulation facilitated by the *risshin* chair may be associated with how students evaluate the meaningfulness and sense of fulfilment of their daily school experiences. Sources of experienced meaning have been shown to include engagement in educational activities, states of flow, mindfulness (Besika et al., 2022) and positive affect (King et al., 2016). Meaningful activities are typically characterized by positive subjective qualities associated with active engagement, and the extent to which individuals engage in such activities is positively related to their sense of meaning (Eakman, 2013).

In the context of schooling, academic activities represent the primary domain requiring sustained engagement over extended periods. The degree to which students can participate actively—physically, emotionall, and cognitively—in these activities may influence their sense of fulfilment in school life. Supporting this perspective, previous research on *risshin* chairs in high school classrooms demonstrated that improved posture was associated with changes in time perception during class, which were linked to higher levels of daily meaningfulness (i.e., perceived fulfilment of the school day; Murakami et al., 2022).

From this perspective, maintaining an optimized posture through an embodied intervention may reduce physical discomfort and effort required for postural control, thereby allowing students to allocate more attentional resources to classroom activities. In addition, an upright posture may function as a bodily affordance that supports a state of readiness and approach‐oriented engagement towards ongoing classroom tasks, which may facilitate students' subjective sense of meaningful involvement in their learning activities. Through the accumulation of engaged learning experiences, students may perceive their school days as meaningful and fulfiling.

### Aims and hypotheses

Based on the 4E cognition framework, this study aimed to replicate and extend Murakami et al. (2022) by implementing a continuous *risshin* chair intervention to examine its impact on junior high school students' posture, class time perception and subjective well‐being including positive affect and daily school fulfilment.

We hypothesized that *risshin* chairs would naturally assist an upright posture during class by acting as somatic scaffolds. This support is expected to reduce the cognitive load required to maintain bodily stability, thereby allowing students to engage in more focused activities. This would be reflected in accelerated perceived class time and reduced task‐unrelated thoughts (i.e., time‐related distraction).

Furthermore, we hypothesized that embodied regulation would enhance both positive affect and daily school fulfilment. Based on prior findings (Murakami et al., 2022), we expected that improved posture via *risshin* chairs would lead to faster class time perception, which may also contribute to students' school fulfilment.

**FIGURE 1:**
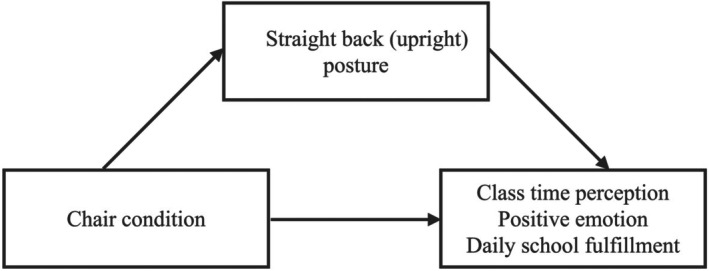
Hypothesized mediation model.

Therefore, we hypothesized that the chair's effects on class time perception and subjective well‐being would be mediated via somatic alignment (i.e., the straight‐back posture; Figure 1). Specifically, we predicted that the *risshin* chair would promote a more extended back, leading to faster time perception, fewer time‐related distracted thoughts, increased positive affect and greater daily school fulfilment. To further explore these pathways, we conducted exploratory serial mediation analyses to examine whether the effects of chair condition on fulfilment were sequentially mediated by upright posture and a second mediator, either class time perception or positive affect.

## MATERIALS AND METHODS

### Participants

Participants were second‐year public junior high school students from Japan's Kansai region. The school has two classes per grade. Recruitment was conducted through discussions with a teacher who was working at the participating school at the time and expressed interest in interventions targeting students' posture. Following discussions with the school, it was requested that second‐year students be selected as the focus of the study owing to the practical ease of implementation. Both classes from the second grade participated in the study.

Four students who attended special education classes, five who did not attend school and one who was absent on all five days of questionnaire completion were excluded. Thus, 48 students (25 girls and 23 boys; *M*_age_ = 13.39 years, *SD* = .49) were finally included. All participants, except those excluded by the criteria mentioned above, experienced the A‐B‐A‐B‐A design, where condition B and condition A involved sitting in *risshin* chairs and regular chairs, respectively. The average attendance rates at each time point were 92.5% at T1, 94.4% at T2, 99.0% at T3, 97.5% at T4, and 94.5% at T5. Participants did not include individuals with locomotor‐related conditions such as spinal or postural disorders. We also told the students to consult their teachers if they had concerns related to their physical posture and were reluctant to participate in the experiment even if they did not have a specific diagnosis.

### Procedures

#### Research design

**FIGURE 2:**
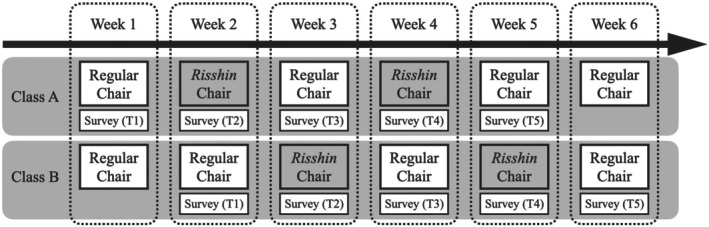
Overview of the intervention. The survey was administered exclusively on the last day of each week.

We employed a within‐participant design. Students alternated between regular and *risshin* chairs in the classroom every week and accordingly experienced their school life (Figure 2). To ensure consistency and address potential concerns about fairness within classes, the school requested that the order of conditions be synchronized within each class. As a result, the starting condition was not randomized at the individual or class level. However, to mitigate biases such as response tendencies influenced by the novelty or excitement of sitting in an unfamiliar chair, we employed an A‐B‐A‐B‐A design instead of a simpler A‐B‐A design. This repeated design allowed students to compare both chair types multiple times, which not only reduced the likelihood that responses would be biased by their initial impressions of the chairs but also enabled us to assess the consistency of condition effects across multiple measurement points, thereby potentially enhancing internal validity. In addition, because chair conditions were alternated weekly and questionnaires were administered only at the end of each week, the carryover effects from the previous week's chair condition were likely minimized.

Considering the students' burden, survey responses were solicited only during homeroom on Fridays (Time 1: T1–Time 5: T5). The survey was created entirely on Google Forms, and students responded via their individual tablets. Additionally, owing to constraints in research funding, limited *risshin* chairs were available. Hence, the study was conducted by alternating conditions between Classes A and B weekly. During the experimental period, an average of 4.4 lessons (50 min per lesson) were offered per day for both classes.

#### Experimental materials

**FIGURE 3:**
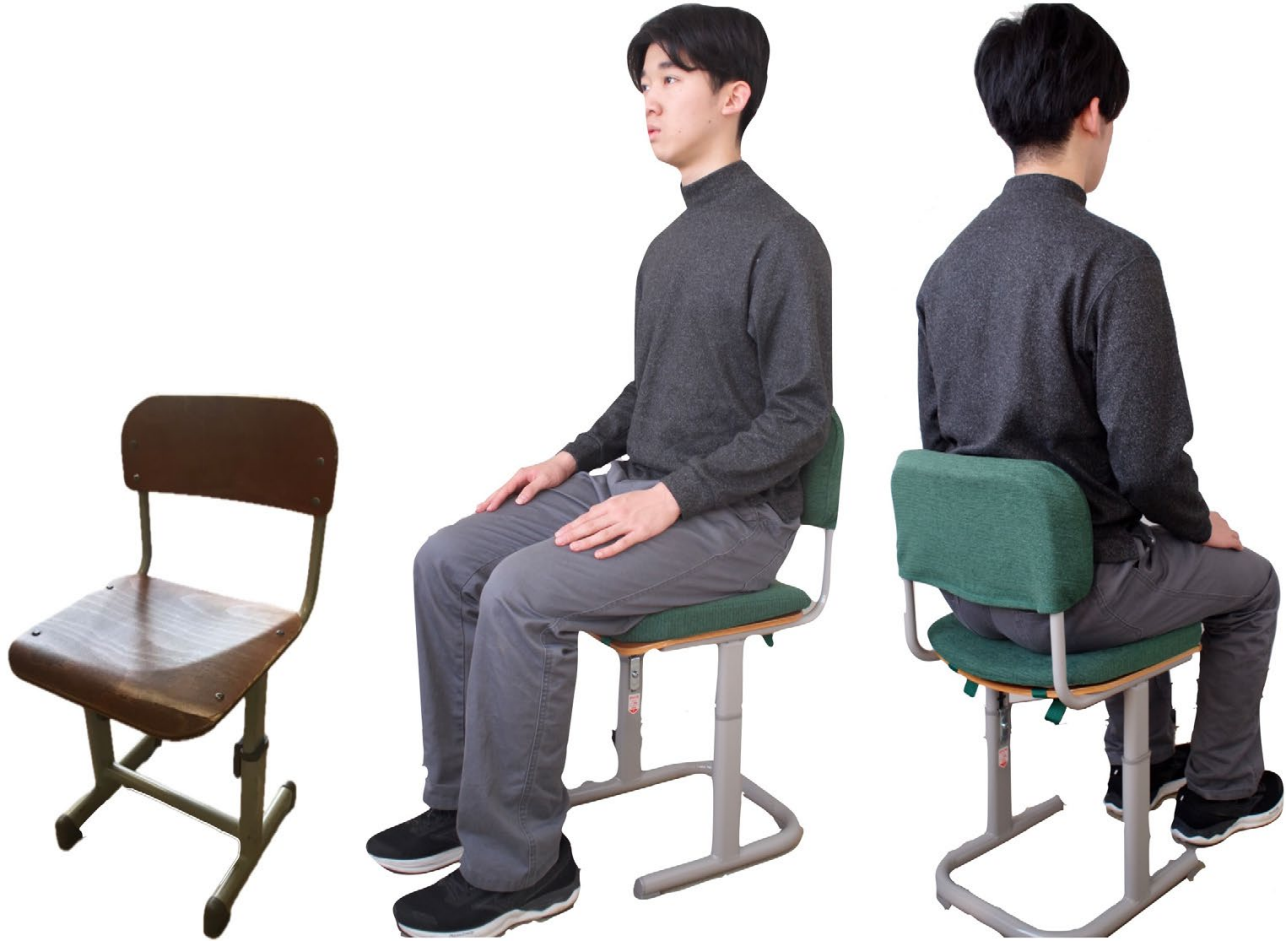
Photographs of the regular chair (left) and *risshin* chair (centre and right) used in the experiment. Owing to restrictions related to rights applications, the image was captured from a position where the *risshin* posture was easily recognizable; however, the exact structure of the cushion was difficult to identify. The model in the photograph was a junior high school student but not a participant in the experiment, and consent for publication was obtained from their guardians.

The regular chairs were part of the school equipment and had been continuously used by students prior to the start of the experiment (Iris Chitose Company, YEC‐601 FW‐LL; Figure 3). The regular chairs were designed with the following dimensions based on the Japanese Industrial Standards: seat width = 360 mm, seat depth = 360 mm, and adjustable seat height = 300–460 mm. The *risshin* chairs utilized a commonly available student chair (Kokuyo Corporation, SCH‐NFU6GN; width = 300 mm, depth = 380 mm, height = 320–460 mm) in Japan, similar in form to regular chairs as their base, to which seat cushions (Okamasa Chair Manufacturing Company, not for sale) were attached to assist with maintaining a *risshin* (upright) posture. Trying to maintain a straight back posture in a regular chair often leads to behavioural characteristics such as increased shoulder tension and a forward tilt of the head; however, this cushion alleviates these physical strains, enabling a more sustainable upright posture. The size and material details of this cushion cannot be disclosed owing to possible patent applications. All students sat deeply in *risshin* chairs of various heights before the intervention to determine the chair size that allowed their feet to naturally rest flat on the floor.

### Measures

#### Posture

A self‐developed questionnaire was used to inquire about participants' postures during class. Items 2–4 were reviewed by two posture research experts to ensure content validity and were previously used in an experiment (Murakami & Sugamura, 2023) targeting second‐year junior high school students. Participants were asked, ‘To what extent do the following statements apply to your posture while sitting in the chair during today's class?’ Statements included the following: (1) ‘I consciously tried to improve my (body) posture,’ (autonomous posture improvement); (2) ‘I sat deeply in the chair,’ (deep seating); (3) ‘My back was straightened,’ (straight back); and (4) ‘Unnecessary tension in my body was released,’ (tension release). Responses were rated on a 7‐point Likert scale that ranged from 1 (not at all) to 7 (very much). Each statement was measured and analysed as a single item. Higher scores indicated a more suitable *risshin* posture.

#### Class time perception

We adapted items from Rutrecht et al.'s study (2021), themselves partly adapted from the Subjective Time, Self, and Space questionnaire (Jokic et al., 2018). Rutrecht et al. (2021) reported significant correlations between these items and a measure of flow during gameplay, suggesting partial support for their construct validity in measuring aspects of subjective time perception. These items were also utilized in Murakami and Sugamura's (2023) study to assess second‐year junior high school students' perceptions of class time over the preceding one to two weeks. Participants were asked, (1) ‘To what extent did you think about the passage of time during today's class? (Have you ever wondered, “How much longer until class is over?”)’ (thinking about time); and (2) ‘How much did today's class feel shorter than it actually was? (Did you feel as if the class was over in a flash while you were awake?)’ (speed of time passage). Responses were rated on a 7‐point Likert scale from 1 (very much) to 7 (not at all) for the former, and from 1 (very long (slow)) to 7 (very short (fast)) for the latter. Higher scores indicated that students experienced fewer time‐related distracted thoughts and perceived the passage of time during class as faster. Each statement was measured and analysed as a single item, consistent with previous research.

#### Subjective well‐being

Positive emotions were measured via the reversed Mood Check List‐Short Form.2 (Hashimoto & Murakami, 2011). This scale has previously demonstrated sufficient reliability and validity (Hashimoto & Murakami, 2011) and been used in mental health research targeting Japanese junior high school students (Miura et al., 2017). Participants were instructed to report their current feelings using items from the ‘pleasantness’ and ‘relaxation’ subscales, each comprising four items. Pleasantness and relaxation included items such as ‘energetic’ and ‘calm,’ respectively. Responses were rated on a 7‐point Likert scale from 1 (not at all) to 7 (very much). Cronbach's alpha for the pleasantness and relaxation scale ranged from .826 to .911 and .721 to .921, respectively.

To assess the daily sense of fulfilment in school life, the Japanese translation of the Daily Meaning Scale (Murakami et al., 2022; Steger et al., 2008), which comprises two items, was employed. Although the original scale was developed to assess the general sense of meaning in one's life, we framed this construct within the context of students' school experiences. Because the questionnaire was administered during the final homeroom session, the instruction ‘Looking back on today’ naturally directed participants' attention to their experience during the school day. Accordingly, the scale was operationalized to measure subjective fulfilment derived from daily school life rather than broader philosophical reflections. The original Daily Meaning Scale has demonstrated sufficient reliability and validity (Steger et al., 2008). While this scale appears to have been used with junior high school students for the first time in the current study, a similar set of items has been used with adolescents of the same age group (Yuan et al., 2024). Additionally, prior to the experiment, item wording was assessed by teachers at the participating school. Participants were asked, ‘Looking back on today, how meaningful did your life feel?’ and ‘Looking back on today, to what extent did you feel your life had purpose?’. Responses were rated on a 7‐point Likert scale that ranged from 1 (not at all meaningful/purposeful) to 7 (very meaningful/purposeful). Cronbach's alpha, measured across five different occasions, ranged from .702 to .897.

#### Preference for chair

Participants were asked regarding both the *risshin* and regular chairs: ‘Would you like to continue using this chair?’ and ‘Were you able to sit comfortably in this chair?’. Responses were rated on a 7‐point Likert scale from 1 (not at all) to 7 (very much). These items were added to the questionnaire only in the final week of each intervention condition (i.e., T4 and T5 for *risshin* and regular, respectively). These items have previously been used with junior high school students (Murakami & Sugamura, 2023).

### Statistical analysis

We employed Bayesian statistical modelling using Hamiltonian Monte Carlo, a variant of Markov Chain Monte Carlo (MCMC), to investigate the relationships between the experimental condition and various outcomes. This approach is well suited for repeated‐measures data in single‐case designs, where increasing the number of data points is inherently difficult. No formal sample size planning was conducted prior to data collection, and Bayesian estimation provides a practical advantage by helping stabilize parameter estimates when sample sizes are small or imbalanced. By incorporating prior distributions, Bayesian methods allow for more stable parameter inference under such conditions (Rindskopf, 2014), particularly relevant for mediation analysis where parameter uncertainty can be substantial.

Four MCMC chains were run with 12,000 iterations each (including 2000 warm‐up iterations), yielding 40,000 post warm‐up samples in total. Convergence diagnostics ($\hat{R}$) were below 1.01 for all parameters. Posterior distributions were obtained using the *brms* package (Bürkner, 2017) with default weakly informative priors (Falk et al., 2024). For each parameter, we reported the posterior median (MED) of samples, 95% highest density intervals (HDI), and the probability that the hypothesis is correct (PHC; Toyoda, 2024), defined as the posterior probability that a parameter is greater than (or less than) zero. For mediation models, indirect effects were computed from the posterior samples by multiplying the relevant path coefficients for each draw, and HDI and PHC were calculated for these derived effects. Bayesian conditional *R* ^2^ values were computed to evaluate model fit. The *bayestestR* package (Makowski et al., 2019) was used to obtain HDIs for each coefficient, and the *performance* package (Lüdecke et al., 2021) was used to compute Bayesian conditional *R* ^2^ and related indices.

Missing data occurred in some repeated measures owing to participant availability (overall missing data rate = 8.38%, range per item = 0%–14.6%). The *TestMCARNormality* function (*MissMech* package; Jamshidian et al., 2014) indicated a near‐singular system, limiting formal missing completely at random testing. Nonetheless, Twisk et al. (2013) reported that, in longitudinal data analysed using mixed models, results from complete‐case analysis and those using multiple imputation are generally comparable, indicating that multiple imputation is not essential in such contexts. Therefore, to reduce computational burden, we proceeded with Bayesian analyses using the complete‐case dataset.

As a preliminary analysis, we fitted Bayesian linear mixed‐effects (BLM) models to assess the effects of *risshin* chairs on posture‐related outcomes (autonomous posture improvement, deep seating, straight back and tension release). Each model included chair condition (0 = regular chair, 1 = *risshin* chair) as a fixed effect. Class (0 = class A, 1 = class B) and gender (0 = boy, 1 = girl) were centred at the grand mean and included as covariates to control for potential confounding effects. Random intercepts and slopes for the chair condition were specified for individual students to account for the nested data structure. The standard deviations of these random slopes ranged from .26 to .94, indicating substantial individual variability in the effect of the chair condition. Interaction terms were not included in the models, as the primary focus was on the main effects of the chair condition.

For the main analysis, we conducted a set of single‐mediator Bayesian mediation models with linear mixed‐effects modelling (BMLM) to examine the indirect effects of chair condition (independent variable: 0 = regular chair, 1 = *risshin* chair) on each psychological outcome. In each model, the straight back posture was specified as the sole mediator, based on prior literature linking upright posture to psychological outcomes. As our intervention specifically targeted upright posture, we focussed solely on this mediator to reduce statistical and interpretational complexity. The dependent variables, tested in separate models, were perceived class time (‘thinking about time’ and ‘speed of time passage’) and subjective well‐being (‘pleasantness’, ‘relaxation’ and ‘daily school fulfilment’). Chair condition was entered as a fixed effect, with class (0 = class A, 1 = class B) and gender (0 = boy, 1 = girl) included as grand mean‐centred covariates. Random intercepts and slopes for the chair condition were specified at the participant level, with the standard deviations of random slopes ranging from .17 to 1.05.

As a supplementary analysis, we tested serial (two‐mediator) BMLMs to explore the mechanisms linking upright posture to daily school fulfilment. Chair condition was the independent variable, straight back posture served as the first mediator, and either perceived class time (‘thinking about time’ or ‘speed of time passage’) or positive emotions (‘pleasantness’ or ‘relaxation’) served as the second mediator. Daily school fulfilment was the dependent variable. Given that the main analysis revealed no significant total or direct effect of chair condition on daily school fulfilment, the direct path from chair condition to daily school fulfilment was omitted to focus on indirect pathways through the sequential mediators. Control variables and random effects were specified in the same manner as in the main models.

R (version 4.3.3; R Core Team, 2024) and R studio (version 2023.12.1 + 402; RStudio Team, 2022) were used for statistical analyses. *BruceR* (Bao, 2024), *psych* (Revelle, 2024), *ggdist* (Kay, 2024), *ggplot2* (Wickham, 2016), and *patchwork* (Pedersen, 2024) packages were also utilized.

### Ethical considerations

Informed consent was obtained from the school principal, guardians and students. Guardians received a detailed explanation based on a pre‐explanatory document that outlined the research overview, voluntary participation and protection of personal information. Consent was obtained via a form that mentioned the names of both the guardians and students. Students received prior explanations based on a request document that detailed the research overview. Furthermore, the teachers at the implementing school carefully explained that participation was voluntary, and all students participated voluntarily with their consent. Additionally, the survey instructions had explicit provisions, such as ‘irrelevant to grades’ and ‘privacy protection ensured,’ to ensure no coercion in responses. This study was approved by the ethics review committee of the affiliated institution (#318).

## RESULTS

### Descriptive statistics

We calculated the mean and standard deviation for each variable at each time point (Table 1).

**TABLE 1:** Descriptive statistics of the variables.

Variables	T1 (*n* = 44)		T2 (*n* = 41)		T3 (*n* = 48)		T4 (*n* = 45)		T5 (*n* = 42)	
	*M*	*SD*	*M*	*SD*	*M*	*SD*	*M*	*SD*	*M*	*SD*
Posture										
Autonomous posture improvement	3.20	1.87	3.83	1.91	3.40	1.76	4.04	1.82	3.83	1.71
Deep seating	4.75	1.81	4.78	1.74	4.56	1.74	4.98	1.60	4.93	1.55
Straight back	3.27	1.72	4.15	1.73	3.33	1.60	4.18	1.57	3.81	1.61
Tension release	3.61	1.78	4.22	1.80	3.73	1.72	4.18	1.64	3.86	1.76
Class time perception										
Thinking about time	2.05	1.18	3.02	1.64	2.88	1.48	3.18	1.71	3.33	1.62
Speed of time passage	2.84	1.41	3.63	1.43	3.46	1.69	3.71	1.47	3.74	1.47
Subjective well‐being										
Pleasantness	3.64	1.25	3.62	1.43	3.34	1.45	3.60	1.42	3.71	1.27
Relaxation	4.45	1.17	4.54	1.41	4.09	1.51	4.29	1.56	4.40	1.51
Daily school fulfilment	3.67	1.71	3.93	1.54	3.68	1.62	3.99	1.52	4.08	1.48

*Note*: T1, T3, and T5 correspond to the weeks in which a regular chair was used by the students, while T2 and T4 correspond to the weeks in which the *risshin* chair was used.

### BLM modelling

The posterior distribution indicated significant positive effects of chair condition on autonomous posture improvement (MED = .449, 95% HDI [.058, .843], PHC = .987, Bayesian conditional *R* ^2^ = .51), straight back (MED = .667, 95% HDI [.213, 1.104], PHC = .998, Bayesian conditional *R* ^2^ = .48), and tension release (MED = .478, 95% HDI [.082, .885], PHC = .990, Bayesian conditional *R* ^2^ = .54), but not on deep seating (MED = .157, 95% HDI [−.207, .509], PHC = .806, Bayesian conditional *R* ^2^ = .46).

### BMLM modelling

#### Outcome: Class time perception

The analysis revealed a significant indirect effect of condition on thinking about time via the straight back posture (MED = .305, 95% HDI [.028, .665], PHC = .991; Table 2). In contrast, the indirect effect on the perceived speed of time passage was not significant (MED = .090, 95% HDI [−.146, .363], PHC = .814). The 95% HDIs for both the total and direct effects in each model included zero.

**TABLE 2:** Results of the Bayesian mediation analysis with linear mixed‐effects modelling.

	Estimation^a^	[95% HDI]	PHC
Thinking about time (*R* ^2^_mediation_ = .48; *R* ^2^_outcome_ = .60)			
Indirect	.305	[.028, .665]	.991
Direct	−.010	[−.411, .398]	.480
Total	.309	[−.042, .667]	.959
Speed of time passage (*R* ^2^_mediation_ = .46; *R* ^2^_outcome_ = .46)			
Indirect	.090	[−.146, .363]	.814
Direct	.182	[−.209, .581]	.828
Total	.284	[−.047, .626]	.952
Pleasantness (*R* ^2^_mediation_ = .42; *R* ^2^_outcome_ = .65)			
Indirect	.363	[.054, .739]	.999
Direct	−.382	[−.792, −.011]	.015
Total	−.011	[−.259, .248]	.464
Relaxation (*R* ^2^_mediation_ = .43; *R* ^2^_outcome_ = .60)			
Indirect	.293	[−.001, .673]	.989
Direct	−.222	[−.663, .174]	.132
Total	.079	[−.189, .354]	.720
Daily school fulfilment (*R* ^2^_mediation_ = .47; *R* ^2^_outcome_ = .69)			
Indirect	.354	[.058, .742]	.997
Direct	−.254	[−.691, .115]	.083
Total	.108	[−.164, .394]	.780

*Note*: The Bayesian conditional *R* ^2^ for the mediation model (*R* ^2^_mediation_) and the outcome model (*R* ^2^_outcome_) are reported. A Bayesian mediation analysis with linear mixed‐effects modelling was conducted using four Markov Chain Monte Carlo chains, each with 12,000 iterations and a 2000‐iteration warm‐up. This resulted in a total of 40,000 post warm‐up samples. All mediation models include the background confounders (class and gender) and specify straight back as the only mediator.

Abbreviations: HDI, highest density interval; PHC, probability that the hypothesis is correct.

^a^ Posterior median (MED) estimation.

#### Outcome: Subjective well‐being

The analysis demonstrated a significant indirect effect of condition on pleasantness via the straight back posture (MED = .363, 95% HDI [.054, .739], PHC = .999). However, the indirect effect on relaxation was not significant (MED = .293, 95% HDI [−.001, .673], PHC = .989). The indirect effect of daily school fulfilment was significant (MED = .354, 95% HDI [.058, .742], PHC = .997). The 95% HDIs for total effects included zero in all three models. For direct effects, only pleasantness had a negative estimate, with its HDI excluding zero.

#### Supplementary analyses

To further investigate the mechanisms linking the experimental condition to the daily school fulfilment, we employed four Bayesian serial mediation models using linear mixed‐effects modelling. These models explored how straight back posture (M1) and various psychological states (M2) serve as serial mediators of the effect of condition (X) on daily school fulfilment (Y).

The serial mediation effects via thinking about time (MED = .071, 95% HDI [−.020, .253], PHC = .953) and speed of time passage (MED = .027, 95% HDI [−.036, .137], PHC = .865) were not statistically supported. In contrast, the models with pleasantness (MED = .333, 95% HDI [.057, .704], PHC = .999) and relaxation (MED = .218, 95% HDI [.007, .539], PHC = .995) as mediators were both statistically supported.

### Preference for *risshin* chair

**FIGURE 4:**
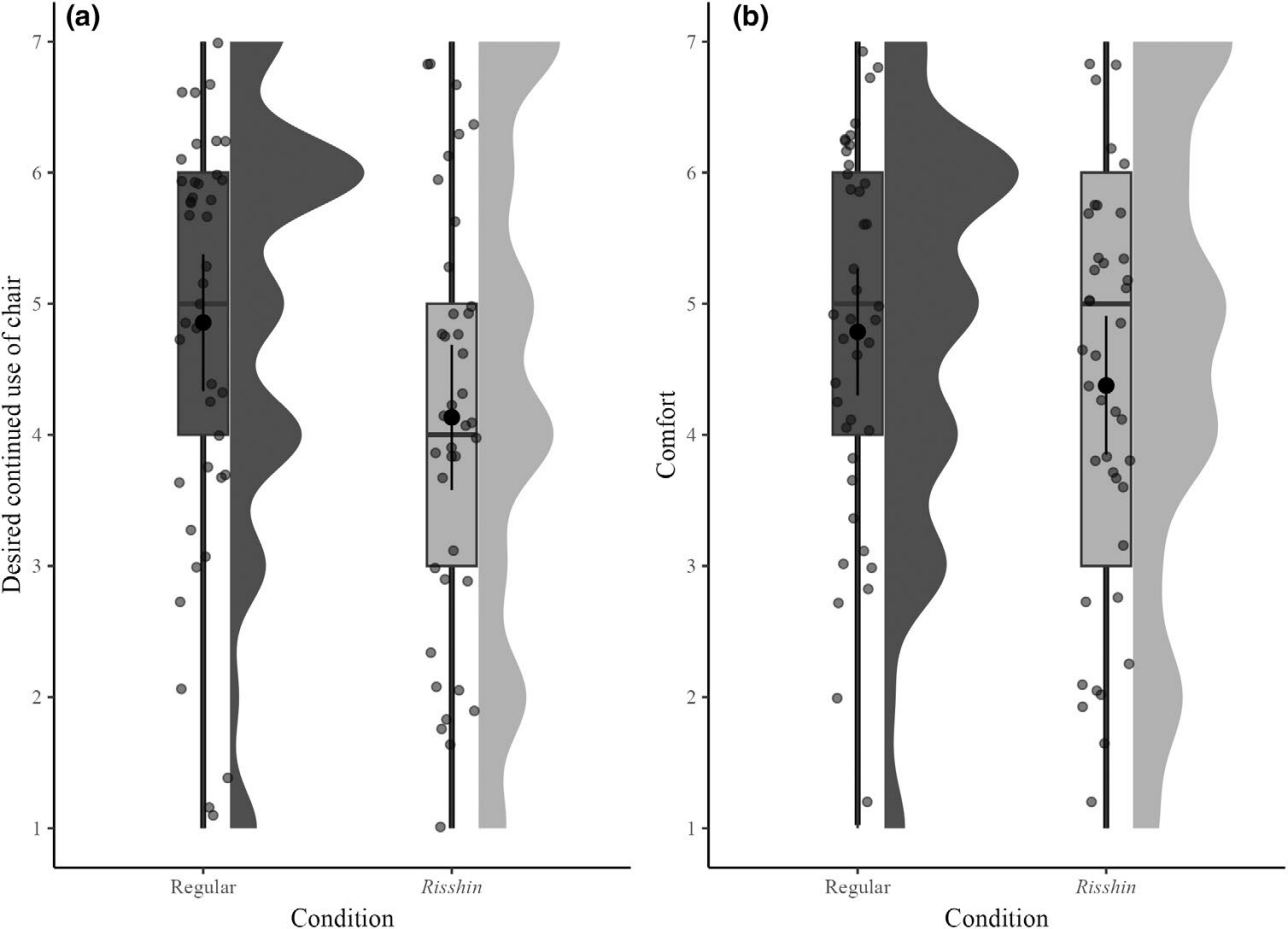
Visualization of the desired continued use of chair (a) and comfort (b) with raincloud plot.

Finally, a paired *t*‐test was conducted to compare desired continued use and comfort level for each chair across the conditions. The difference in the desired continued use of the chair between the regular (*M* = 4.83, *SE* = .26) and *risshin* chair conditions (*M* = 4.05, *SE* = .29) was not statistically significant (*t* = 1.59, *df* = 40, *p* = .119, *d* = .25, 95% confidence interval [CI; −.06, .56]; Figure 4). Furthermore, no statistically significant difference (*t* = .91, *df* = 40, *p* = .369, *d* = .14, 95% CI [−.17, .45]) was observed in chair comfort between the regular (*M* = 4.76, *SE* = .24) and *risshin* chair conditions (*M* = 4.34, *SE* = .28).

## DISCUSSION

We aimed to replicate and extend Murakami et al.'s (2022) study and examined the effects of an intervention via a postural support chair on junior high school students' physical posture, class time perception, and subjective well‐being. We predicted that the back would be more extended in the *risshin* than regular chair condition, which would in turn lead to faster time perception, fewer time‐related distracted thoughts, improved positive emotions, and enhanced daily school fulfilment. BLM and BMLM revealed that the effects of the *risshin* chair on these outcomes partially supported the hypotheses, except for a few indicators. These results suggest that the *risshin* chair functions as an extended regulatory resource, stabilizing students' embodied foundations, and facilitating more effective self‐regulation during classroom activities.

### Effects of *risshin* chair on posture

Compared with the regular chair condition, the *risshin* chair condition was associated with higher predicted values for ‘autonomous posture improvement’, ‘straight back’ and ‘tension release’, suggesting its effectiveness in improving students' physical posture. This effect persisted even after controlling for gender and class differences as well as random effects for participants. Based on the PHC values, the probability that these posture‐related variables have a positive effect was estimated at 98.7%–99.8%. Murakami et al. (2022) revealed postural improvement in high school students; however, we extended these findings to early adolescents in public middle schools. Given the physical strain of prolonged sitting (Caromano et al., 2015), tools that assist posture through environmental adjustments, such as the *risshin* chair, are beneficial.

However, the model did not show a significant effect on ‘deep seating’, potentially because students continuously adjusted their sitting positions based on their physical condition. The PHC for this variable was 80.6%, indicating a relatively lower probability of a positive effect compared with the other posture‐related variables. Considering the improvement in other posture‐related outcomes, the *risshin* chair seemed to stretch the spine and relieve excess muscle tension without forcing the user to maintain a fixed sitting position.

### Effects of *risshin* chair on class time perception and subjective well‐being via a straight back

First, the mediation model with class time perception was partially supported. Specifically, the indirect effect of posture on thinking about time was significant (PHC = 99.1%), whereas its effect on the perception of time passing quickly was less robust (PHC = 81.4%). In Murakami et al. (2022), which utilized only ‘time acceleration’ as a concentration index, private high school students likely exhibited higher baseline engagement, potentially enhancing posture‐induced immersion. In contrast, the present study involved public junior high school students with more varied academic motivation, possibly limiting the impact of posture on such time distortion. Nevertheless, the significant reduction in off‐task thoughts unrelated to the class content represents a novel contribution. From the 4E and bioenergetic perspectives, this suggests that the *risshin* chair functions as a somatic scaffold, optimizing body segment alignment (Canales et al., 2010). By facilitating a ‘straight back’ alongside ‘tension release’, the chair likely minimized the hidden compensatory effort required for postural stability (Guimond & Massrieh, 2012). This postural efficiency suggests a reduction in ‘hidden’ somatic demands, which may have helped students shift their attention away from bodily regulation and to remain more consistently situated in classroom tasks.

Second, the mediation model revealed significant indirect effects of upright posture on pleasantness (PHC = 99.9%) and daily school fulfilment (PHC = 99.7%) but not on relaxation (PHC = 98.9%). Thus, this hypothesis was partially supported. This aligns with the finding that an upright posture promotes an alert, activated state (Nair et al., 2015) and positive emotions (Awad et al., 2021; Haruki & Suzuki, 1994). From the perspective of the self‐perception hypothesis (Bem, 1972), students may have subconsciously monitored their supported upright posture and inferred a sense of readiness and positive affect. Our exploratory sequential mediation analysis further clarified this mechanism: the pathway to school fulfilment was supported only when mediated by positive affect (e.g., pleasantness), whereas cognitive variables, such as time perception, did not show significant sequential mediation. This finding may partially align with prior research, suggesting that high‐arousal positive affect exerts a stronger momentary impact on daily meaningfulness among adolescents (Chu et al., 2020). This suggests that, for adolescents, school fulfilment is grounded more in immediate somatic‐affective experiences than in reflective, cognitively mediated shifts in attention. Within the 4E framework, these results imply that a ‘straight back’ functions as a somatic scaffold not only for focus but also for emotional appraisal, allowing students to enact a more vivid and meaningful school experience through high‐arousal positive emotions. However, the negative direct effect of the condition on pleasantness suggests that the mere presence of the *risshin* chair is insufficient and that the psychological benefits are contingent on the actual maintenance of an upright posture.

Overall, the absence of significant positive total and direct effects in our mediation models requires careful interpretation. Significant mediation effects can exist even without a clearly identifiable total effect under certain conditions (O'Rourke & MacKinnon, 2018). In our study, the positive effects on thinking about time, pleasantness, and daily school fulfilment were not simply due to the use of the chair itself, but were specifically mediated by the ‘upright posture’ it facilitated. This suggests that the observed benefits emerge through behavioural and physiological improvements supported by the chair, consistent with prior research emphasizing the psychological significance of upright posture. These findings underscore the importance of investigating mediating pathways in posture‐focused interventions to better understand their broader psychological impact. From this perspective, an upright posture may function as a bodily affordance that supports engagement in classroom learning activities, and interventions targeting this posture may contribute to improvements in attentional stability and subjective well‐being.

### Preference for chair

No significant disparity was observed in desired continued use or comfort level between *risshin* and regular chairs. Murakami et al. (2022) indicated a notably high average for the desired continued use of *risshin* chairs, whereas this value was relatively low in our study. Murakami et al. (2022) targeted students in advanced high school classes, which potentially led to an elevated motivation towards classes as a whole and thus resulted in students favouring chairs that assisted posture. Alternatively, in our study, students were required to alternate chairs weekly. Furthermore, such procedural inconvenience might have exerted a non‐negligible influence on the assessments of the chairs' desired continued use and comfort level. When introducing *risshin* chairs into classes with pre‐adolescent students, it is necessary to consider individual differences in student preferences for these chairs.

### Practical implications

Although short‐term, the *risshin* chair improved specific aspects of students' physical posture, class time perception, and subjective well‐being during school compared with regular chairs. Thus, posture‐supportive seating such as the *risshin* chair could serve as a somatic scaffold that enhances classroom engagement and comfort. Upright posture has been linked to better cognitive function (Inagaki et al., 2018) and academic performance (Weisfeld & Beresford, 1982), as well as positive emotions, which predict academic outcomes such as mathematics test scores (Ohtani & Nakaya, 2013). While this study does not directly address academic outcomes, the results highlight the potential for posture interventions to impact both physical and cognitive aspects of learning. Future research should explore the broader educational benefits of such interventions.

We detected the effects of the *risshin* chair, even after accounting for individual differences, gender and class. This suggests that posture‐supportive seating may be an effective universal design strategy that is especially beneficial for students who require both physical and mental support. Teachers should carefully consider classroom seating arrangements and prioritize ergonomic designs that support both physical health and effective learning.

### Limitations and further research

Regarding methodological limitations, we relied solely on self‐reported questionnaires, including some measures without established reliability and validity for junior high school students. While subjective ratings are essential for capturing posture‐related bodily sensations, class time perception and well‐being, the possibility of response bias cannot be ruled out. Future research should incorporate objective and physiological measures, such as electromyography, salivary cortisol and standardized behaviour/posture assessments (e.g., Mahar et al., 2006; Otsui et al., 2006), to more accurately evaluate the effects of the *risshin* chair. Posture estimation utilizing photographic data or motion capture, as well as experience sampling via wearable devices, could further improve the precision of both physical and psychological data. Integrating these dynamic and objective indicators with subjective ratings would strengthen the validity of the *risshin* chair intervention's effects.

Second, the setting was a relatively small rural public middle school, and we did not conduct an a priori power analysis owing to both technical and practical reasons. Technically, our single‐case design lacks well‐established methods for formal sample size estimation, and practically, we were limited to the number of students available in the two classrooms that made up the entire grade level at the cooperating school. Additionally, teacher workload and limited intervention resources made expanding the sample infeasible. To mitigate potential concerns about small sample size, we used BLM models, which can provide more stable estimates than frequentist methods. We used weakly informative priors in our analysis and the models converged appropriately, yielding interpretable and robust estimates. Moreover, the posterior distributions derived from this study can serve as prior information for future research, contributing to the accumulation of evidence in this field. Future research should address this limitation by incorporating power analysis during the planning phase to ensure robust study designs and reliable effect detection. Furthermore, previous research on Japanese junior high schools indicates that larger school sizes correlate with decreased class cohesiveness and orderly behaviour (Mizuno, 2008). Consequently, the distribution of class concentrations may vary in larger schools. Future studies should explore the effects of *risshin* chairs in diverse schools and class sizes to better understand their impact.

Looking towards future research, it is essential to extensively examine the broader effects of the *risshin* chair. These include potential impacts on both mindfulness and interpersonal interactions among students and between students and teachers. Our intervention increased students' awareness of the need to improve their posture and reduced time‐related distractions during class. This finding suggests an enhancement in bodily awareness and attention to the present moment, which may indicate a heightened state of mindfulness. Furthermore, as discussed by Murakami et al. (2022), if students are able to concentrate in classes with better posture, the quality of explanations and responses from teachers to students may improve. Additionally, if students observe their peers maintaining an upright posture during lessons, they may adjust their own posture to align with their peers' attentive behaviour. Such interpersonal interactions within the classroom represent potential intervention effects that warrant further investigation. This aligns with the 4E perspective that cognition is not only embodied but also socially embedded and extended (Ferreira & Ineson, 2026). To assess these impacts, future studies could employ observational methods to record and analyse interactions among students and between students and teachers.

Future research should examine the effect of posture/embodied education. Posture education is to learn that differences in physical posture can affect various aspects, such as emotions, cognitive functions and impressions of others. We demonstrated that a *risshin* chair had positive effects on several posture‐related variables. However, it is crucial for learners to be acutely aware of their posture while learning. Therefore, integrating contemplative practices in posture education alongside the use of the *risshin* chair may help students enhance their awareness of bodily sensations and posture adjustments. From the perspective of preventive education for mental and physical health, developing posture education and examining its long‐term effects are future directions.

## CONCLUSION

We aimed to replicate and extend previous findings and examine the effects of a *risshin* chair on physical posture, class time perception and subjective well‐being among junior high school students using a Bayesian analytical framework. The *risshin* chair significantly improved posture, which included autonomous posture improvement, a straight back and tension release, even after accounting for individual differences. It also reduced time‐related distractions during class and enhanced specific aspects of well‐being, such as pleasantness and daily school fulfilment, through a straight back. However, not all outcomes showed significant effects—for example, perceived class time and relaxation were not significantly influenced. Moreover, in most models where significant indirect effects were observed, the total and direct effects did not show statistically supported positive associations. These findings suggest that the observed benefits are primarily attributable to improved upright posture facilitated by the chair, which plays a central role in supporting certain dimensions of physical regulation, attentional focus and emotional experience during school. This study highlights the potential of optimizing students' somatic foundations as an environmental approach to support school adaptation. Future research should incorporate physiological measures and explore diverse school settings to further validate these effects.

## AUTHOR CONTRIBUTIONS

**Yusuke Murakami:** Conceptualization; methodology; software; data curation; formal analysis; validation; investigation; funding acquisition; writing – original draft; visualization; project administration; resources; writing – review and editing. **Takashi Akiyama:** Methodology; software; validation; formal analysis; writing – review and editing; visualization; data curation. **Genji Sugamura:** Conceptualization; methodology; writing – review and editing; investigation; validation; supervision.

## CONFLICT OF INTEREST STATEMENT

The authors declare that there are no conflicts of interest regarding the publication of this paper.

## Data Availability

Data supporting the findings of this study are available upon request from the corresponding author. The data are not publicly available due to privacy or ethical restrictions.
